# Successful treatment of invasive aspergillosis caused by *Aspergillus parafelis* in a kidney transplant recipient

**DOI:** 10.1016/j.mmcr.2020.10.001

**Published:** 2020-10-17

**Authors:** Antonia Calvo-Cano, Eugenio Garduño-Eseverri, Ana Alastruey-Izquierdo, Román Hernández-Gallego, Rocío Martínez-Gallardo, Francisco Félix Rodríguez-Vidigal

**Affiliations:** a Unidad de Patología Infecciosa, Hospital Universitario de Badajoz, Badajoz, Spain; b Servicio de Microbiología, Hospital Universitario de Badajoz, Badajoz, Spain; c Servicio de Micología, Centro Nacional de Microbiología, Instituto de Salud Carlos III, Majadahonda (Madrid), Spain; d Servicio de Nefrología, Hospital Universitario de Badajoz, Badajoz, Spain

**Keywords:** *Aspergillus*, *Aspergillus parafelis*, Invasive aspergillosis, Kidney transplant, Azole-resistant aspergillosis

## Abstract

Invasive aspergillosis (IA) is associated with a high mortality rate in kidney-transplant recipients. Azole-resistance is increasing in *Aspergillus fumigatus*. We report a clinical case of a kidney-transplant recipient with cerebellar and pulmonary aspergillosis caused by azole-resistant *Aspergillus parafelis* (molecular identification through β-tubulin sequence). The patient experienced an effective resolution after three surgical procedures and associated antifungal therapy. This case highlights that azole-resistant aspergillosis should be considered in every patient with IA as long as susceptibility testing results are not known. Therefore, in selected patients with IA and central nervous system involvement, empirical combination antifungal therapy could be considered.

## 1 Introduction

Invasive aspergillosis (IA) has become an important cause of life-threatening infections in immunocompromised hosts. In kidney transplant recipients, IA has one-year incidence of 0.65%, and it is associated with a high 12-weeks mortality rate that ranges from 16% to 39% [[1], [2], [3]]. *Aspergillus fumigatus* is the most common agent and monotherapy with voriconazole is the elective therapy, including clinical forms that affect central nervous system (CNS), according to the latest IDSA Guidelines [4].

In recent years, resistance to triazoles in *Aspergillus fumigatus* has emerged worldwide [5]. In some areas of Europe, IA caused by azole-resistant *A. fumigatus* (ARAF) can come to represent near a fifth of cases and shows an exceedingly high mortality [6]. Azole monotherapy should be avoided if resistance is suspected [7] and empirical combination therapy (voriconazole plus an echinocandin or liposomal amphotericin B (L-AmB)) is proposed in regions with environmental azole resistance rates of ≥10%, choosing L-AmB as core therapy if CNS is involved [4,8,9].

Two different mechanisms have been described as responsible of ARAF: 1) acquired resistance during prolonged azole therapy [10], or linked to environmental fungicide exposure [11], and 2) cryptic *Aspergillus* species belonging to the section *Fumigati*, which display some level of intrinsic resistance to antifungal drugs [12,13]. These strains are often misidentified as *A. fumigatus* by conventional morphological analysis and by Internal Transcribed Spacer sequencing.

Here we describe a clinical case of a kidney-transplant recipient with IA caused by *A. parafelis*, an intrinsic azole-resistant cryptic species of *Aspergillus* section *Fumigati*. The patient was successfully treated. Our objective is to show the complex management of this case with repeated surgical interventions and empirical antifungal combination therapy.

## 2 Case

**Fig. 1:**
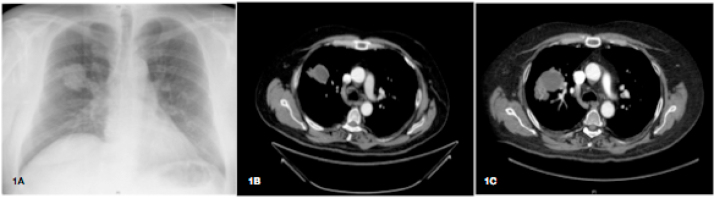
1A: Chest X-ray on admission. 1B: Chest CT scan on admission. 1C: Chest CT scan on day +100, with no response to antifungal therapy.

**Fig. 2:**
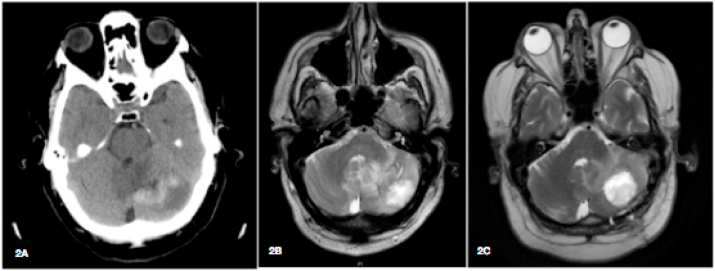
2A: Brain CT scan on day +7. 2B: Magnetic resonance imaging on day +10. 2C: Magnetic resonance imaging on day +100 (recurrence after resection on day +81).

A 56-year-old male who received a kidney transplant of deceased-donor 9 months earlier (day −270), was admitted to our hospital (day 0). He suffered from mild headache and fever. On physical examination, lungs showed hypophonesis in right middle field; no other abnormalities were detected. Chest CT scan showed an irregular mass of 58 × 40 mm in diameter, in the right upper lobe (Fig. 1A and B). The white-cell count was 1500 per cubic millimeter, with 350 granulocytes; C-reactive protein was 147 mg/L. He had diabetes mellitus type 2, he worked in agricultural activities, still performed as a hobby, and he was living with his wife and one cat. Basiliximab had been used as induction therapy when transplanted, and tacrolimus (trough target 6 ng/ml), mycophenolate mofetil (500 mg bid), and prednisone (5 mg qd) had been used for maintenance. On day −60, he developed cytomegalovirus (CMV) enterocolitis, treated effectively with valganciclovir. On day −45, mycophenolate mofetil and valganciclovir were stopped due to neutropenia. At admission, empirical treatment with intravenous voriconazole (400 mg bid), cefepime (2 g tid) and trimethoprim/sulfamethoxazole (320/1600 mg tid) was initiated. Therapeutic Drug Monitoring (TDM) of voriconazole showed sufficient exposure: 5 μg/ml on day +13, 3,6 μg/ml on day +21. Valganciclovir was reintroduced due to CMV load increased. Frontal headache was increasing, and the cranial CT-scan (day +7) showed a cerebellar lesion (Fig. 2A), confirmed by MRI brain on day +10 (Fig. 2B). Diagnosis of invasive pulmonary aspergillosis with CNS involved was made on day +21, based on: 1) a positive galactomannan test (10,25) both in plasma and in bronchioalveolar lavage [4], 2) a positive *Aspergillus fumigatus* complex PCR result in bronchioalveolar aspirate, 3) histological septate hyphae and dichotomous branching in puncture aspiration of the lung nodule, and 4) culture of *Aspergillus* sp. in cerebrospinal fluid (CSF). No brain biopsy was performed, susceptibility testing was not available for the species recovered from CSF.

Defervescence and radiological stability, in both pulmonary and CNS localizations, were obtained on day +35, although galactomannan plasma levels decreased little and remained positive (from 10,25 to 6). Undetectable plasma CMV load and normal neutrophils counts were achieved before discharge. On day +52 the patient was sent home with oral voriconazole monotherapy (300 mg bid) achieving a plasma voriconazole concentration of 3,8 μg/ml. Species identification, as well as antifungal susceptibility testing, was pending from reference laboratory.

On day +72, the patient was readmitted at our hospital due to hydrocephalic symptoms. A cranial CT-scan showed an increase in size of cerebellar lesion and surrounding edema, causing a collapse of IV ventricle. The patient underwent an external ventricular drain on day +72 and surgical excision of cerebellar lesion was performed (day +81), confirming positive fungal culture for *Aspergillus* sp. in both CSF and in cerebellar tissue culture. Susceptibility testing from this sample was requested to the reference laboratory. Chest CT-scan was repeated (day +100) and showed no reduction in sized of the upper right lobe lesion, but multiple disseminated nodular lesions had appeared (Fig. 1C). Serum galactomannan remained high (>5).

Results from reference laboratory were received on day +110. The strain was identified by morphological methods as *Aspergillus fumigatus species complex* and confirmed by ITS sequence. β-tubulin sequence had 99.2% similarity with the β-tubulin sequence of the type strain of *Aspergillus parafelis* (GenBAnk accession number: KJ914692) and 92.8% of the type strain of *Aspergillus felis* (GenBank accession number JX021700) and it was therefore identified as *Aspergillus parafelis.* The strain was stored in the collection of filamentous fungi of the Department of Mycology of the National Centre of Microbiology and was assigned the strain number CNM-CM9239.

Using EUCAST methodology, susceptibility testing showed the following Minimal inhibitory concentration (MIC): amphotericin B: 2 mg/L, itraconazole: >8 mg/L, voriconazole: >8 mg/L, posaconazole: 1 mg/L, Isavuconazole 8 mg/L, terbinafine: 2 mg/L, and Minimal Effective Concentration (MEC): caspofungin (MEC): 4 mg/L and anidulafungin (MEC): 0.25 mg/L.

Combination antifungal regimen was started and included inhaled and systemic (8 mg/Kg/day) L-AmB, and flucytosine (3 g tid). Despite two weeks on this medical therapy, cerebellar lesion reoccurred (day +100) and no response was achieved in reducing the lung lesions (Fig. 1, Fig. 2C). Therefore, on day +120, the patient underwent a second neurosurgical procedure and on day +130 a right superior lobectomy was performed. After recovery, a multidisciplinary decision of discontinuation the immunosuppression was made. He was discharged on day +150, receiving prednisone (10 mg daily) and monthly intravenous immunoglobulin (500 mg/Kg). As outpatient maintenance antifungal regimen, dual therapy was prescribed, with intermittent doses of micafungin (350 mg iv, twice-weekly) and oral posaconazole (300 mg daily).

On day +180, posaconazole was stopped because of abdominal pain and oral intolerance. Isavuconazole was chosen for continuing outpatient therapy (200 mg bid), together with intermittent micafungin (350 mg iv, twice-weekly). Achieved plasma levels of isavuconazole were between 4 and 6 μg/ml (MIC 8 mg/L) with acceptable tolerability.

**Fig. 3:**
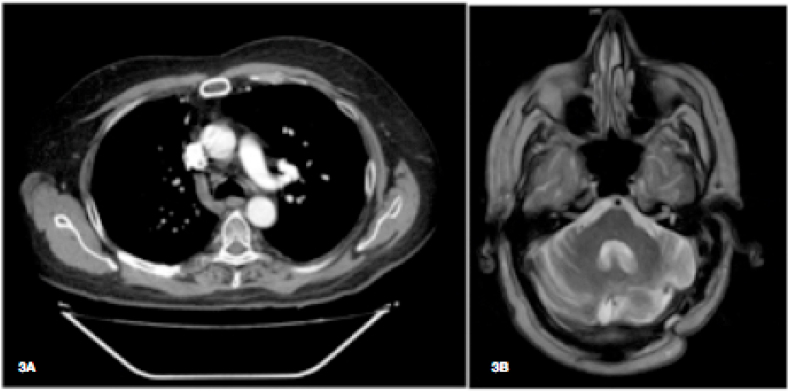
Chest CT scan (3A) and brain magnetic resonance imaging (3B) one year after last surgical procedures.

One year after last surgical procedure (day +500), antifungal treatment was stopped: the patient was in good health, with galactomannan levels <0,5, without signs of recurrence on imaging (Fig. 3A and B). After six months of follow-up without treatment (day +680), he showed no clinical, serological or radiological signs of recurrence of IA. He was on prednisone (10 mg qd) only at this time and graft function stabilized on 1.6 mg/dl creatinine (CKD-EPI: 47.5 ml/min/1.73 m^2^).

## 3 Discussion

The case presented is a late-onset IA episode in a kidney transplant recipient, with pulmonary and CNS involvement, successfully treated despite intrinsic azole-resistance of the causative *Aspergillus* strain. Two factors could explain delayed clinical onset of IA: immunomodulating CMV infection with neutropenia and mold exposure from gardening or caring his cat. Although there is no evidence of transmission from felines to human, *Aspergillus parafelis* has been identified as a pathogen in cats [14]. To our knowledge, this is the first report of human IA caused by *A. parafelis*. The most common cryptic species in *Aspergillus* section *Fumigati* associated with clinical disease in humans are *A. lentulus*, *A. thermomutatus*, and members of the *A. viridinutans* complex. Out of the 10 species within this complex, six have been identified as pathogens in humans and animals. *A. parafelis*, one of them, was firstly identified from human oropharyngeal exudate at National Center for Microbiology, Spain, in 2004 [14].

This case report illustrates the challenges of managing an IA in the current era of emerging species and resistance.

Susceptibility testing for isolated *Aspergillus* spp. is not performed in all laboratories. Hence, the rate of azole-resistance in our environment is probably underestimated and implies a selection bias: just clinical samples from refractory cases of IA are usually referred to the National Center for Microbiology. The same occurs with definitive species identification, which requires specific sequencing analyses of the β-tubulin or calmodulin genes. Mass spectrometry (maldi-tof) could be proposed as a rapid option, able to discriminate *A. fumigatus* from cryptic species. The fact that *Aspergillus* section *Fumigati* includes cryptic species with intrinsic azole-resistance, should make clinicians suspicious about the assumed susceptibility to azoles. Molecular identification at species level should be aimed for, especially when the patient is not responding to first line antifungal treatment.

The receipt of results of susceptibility test from external laboratories may imply a delay of several weeks in certain hospitals. In those circumstances, there may be a need to cover empirically the possibility of azole-resistance, especially if the CNS is involved. Regarding our patient, data of failure with voriconazole therapy was obtained at the same time as the MIC results were available. The offered salvage treatment included a combined surgical approach with combination antifungal therapy following expert recommendations. Once recovered, the choice of appropriated maintenance outpatient therapy was not easy: we used a regimen including two antifungal classes, for at least one year and with good CNS penetration. The main obstacles were high MIC for all antifungals available, no possibility of parenteral domiciliary service in our region, and the adverse events probably related to posaconazole administration. The chosen regimen included micafungin due to its pharmacokinetic properties that allow parenteral administration on alternate days in an outpatient setting, despite its low penetration into the CNS. Isavuconazole seems to show good CNS penetration, but in our patient, achieved plasma levels were lower than expected. More pharmacological data are needed to clarify echinocandins role for IA as alternative therapy in azole-resistant cases [15], and probably new drugs in the pipeline (olorofim, VL-2397) will fill the gap in our therapeutic options as well as for use in outpatient settings [16].

As clinical data on the treatment of cryptic *Aspergillus* species is limited and recommendations for azole-resistant IA are not well established, this case highlights the importance of species identification and susceptibility testing even in patients with no previous exposure to azoles. In severely-affected patients with IA, especially if the CNS is involved, it is worth to consider covering empirically the possibility of azole-resistance with combination antifungal therapy, and explore surgical options to manage disseminated IA.

## Declaration of competing interest

There are none.
